# Identification of a Nomogram from Ferroptosis-Related Long Noncoding RNAs Signature to Analyze Overall Survival in Patients with Bladder Cancer

**DOI:** 10.1155/2021/8533464

**Published:** 2021-08-26

**Authors:** Yuanshan Cui, Zhongbao Zhou, Yumeng Chai, Xuanyan Che, Yong Zhang

**Affiliations:** ^1^Department of Urology, Beijing Tiantan Hospital, Capital Medical University, No. 119 South 4th Ring West Road, Fengtai District, Beijing 100070, China; ^2^Department of Urology, The Affiliated Yantai Yuhuangding Hospital of Qingdao University, Yantai, Shandong, China

## Abstract

**Purpose:**

This study aimed to establish a nomogram to predict the overall survival (OS) of patients with bladder cancer (BC) by ferroptosis-related long noncoding RNAs (FRlncRNAs) signature.

**Methods:**

We obtained FRlncRNAs expression profiles and clinical data of patients with BC from the Cancer Genome Atlas database. The patients were divided into the training set, testing set, and overall set. Lasso regression and multivariate Cox regression were used to establish the FRlncRNAs signature, the prognosis of each group was compared by Kaplan–Meier (K-M) analysis, and the receiver operating characteristic (ROC) curve evaluated the accuracy of the model. The Gene Set Enrichment Analysis (GSEA) was used for the visualization of the functional enrichment for FRlncRNAs. The databases of GEPIA and K-M Plotter were used for subsequent functional analysis of major FRlncRNAs.

**Results:**

Thirteen prognostic FRlncRNAs (LINC00942, MAFG-DT, AL049840.3, AL136084.3, OCIAD1-AS1, AC062017.1, AC008074.2, AC018653.3, AL031775.1, USP30-AS1, LINC01767, AC132807.2, and AL354919.2) were identified to be significantly different, constituting an FRlncRNAs signature. Patients with BC were divided into low-risk group and high-risk group by this signature in the training, testing, and overall sets. K-M analysis showed that the prognosis of patients in the high-risk group was poor and the difference in the subgroup analyses was statistically significant. ROC analysis revealed that the predictive ability of the model was more accurate than traditional assessment methods. A risk score based on FRlncRNAs signature was an independent prognostic factor for the patients with BC (HR = 1.388, 95%CI = 1.228–1.568, *P* < 0.001). Combining the FRlncRNAs signature and clinicopathological factors, a predictive nomogram was constructed. The nomogram can accurately predict the overall survival of patients and had high clinical practicability. The GSEA analysis showed that the primary pathways were WNT, MAPK, and cell-matrix adhesion signaling pathways. The major FRlncRNAs (MAFG-DT) were associated with poor prognosis in the GEPIA and K-M Plotter database.

**Conclusion:**

Thirteen prognostic FRlncRNAs and their nomogram were accurate tools for predicting the OS of BC, which might be molecular biomarkers and therapeutic targets.

## 1. Introduction

Bladder cancer (BC) is one of the most common cancers worldwide, with nearly 430,000 newly diagnosed patients each year [1]. Approximately 90% of BC belong to urothelial carcinoma according to the histological origin [2]. However, unlike other tumors with a single direction of progression, BC is divided into two types, including nonmuscle invasive bladder cancer (NMIBC) and muscle invasive bladder cancer (MIBC) [3]. The two are not different just in the depth infiltration but have distinctly different characteristics and outcomes [4]. The obvious characteristic of NMIBC is high local recurrence rate [5]. Although NMIBC can be treated by transurethral resection, about 20% of patients with NMIBC progress to MIBC within 5 years, followed by lymphatic metastasis and distant organ metastasis [6, 7]. Although the patients undergo radical cystectomy, the 5-year survival rate of patients with MIBC is still less than 50% [8]. Therefore, it is very meaningful to identify molecular markers of tumor progression and early metastasis.

Ferroptosis, as iron-dependent programmed cell death, is mainly characterized by lipid peroxidation [9]. With the development of molecular mechanism research, ferroptosis has been proved to be involved in a variety of important pathophysiological processes, mainly including the occurrence of cancer and the formation of ischemia-reperfusion injury and neurodegenerative diseases [10]. Thus, modulating ferroptosis in tumor cells may be a novel therapeutic modality [11]. An increasing number of studies support the involvement of ferroptosis in the pathophysiology of BC development and progression [12, 13].

Long noncoding RNAs (lncRNAs) were found to play a wide range of roles in a variety of important biological processes, including cell proliferation and differentiation, regulation of gene expression, RNA translation, and regulation of microRNAs [14, 15]. In BC, ferroptosis had dual and contradictory roles in the process of tumorigenesis, but the exact mechanisms that lead to ferroptosis during cancer have not been clearly studied [16]. Ferroptosis-related long noncoding RNAs (FRlncRNAs) can be involved in the invasion, metastasis, prognosis, and chemoresistance of BC via modulating ferroptosis. The FRlncRNAs expression profiles of TCGA database were not performed to explore novel biomarkers for forecasting the prognosis of BC. Therefore, we aimed to utilize TCGA database to establish FRlncRNAs signature and seek new biomarkers to predict the prognosis of patients with BC.

## 2. Materials and Methods

### 2.1. Data Collection

We followed the methods of Dr. Zhou et al. [15]. We obtained RNA sequencing (RNA-seq) of patients with BC from The Cancer Genome Atlas (TCGA, https://portal.gdc.cancer.gov/) database. The inclusion criteria were as follows: (a) patients were diagnosed as BC; (b) patients had complete lncRNA expression profiles and clinical data. According to the inclusion criteria, 414 patients were included in our study. In addition, the clinical data (mainly including age, gender, pathological stage, TNM stage, survival status, survival time, and survival prognosis) were also downloaded from TCGA. We excluded the patients who had a follow-up time of less than 30 days and incomplete RNA-seq and clinical data. The study did not need the approval of the ethics committee because the data involved in this study were all from TCGA database and strictly complied with TCGA guidelines (http://cancergenome.nih.gov/abouttcga/policies/publicationguidelines).

### 2.2. Identification of FRlncRNAs and Differentially Expressed Genes

We obtained the profiles of lncRNAs from all the RNA-seq data and standardized the RNA-seq data by log2 transformation. A total of 14,142 lncRNAs were isolated from the TCGA-BLCA dataset. The Gene Set Enrichment Analysis (GSEA, http://www.gsea-msigdb.org/gsea/index.jsp) was used to download the list of 60 ferroptosis-related genes (FRGs), of which 59 FRGs were expressed in BC. Pearson analysis was used to estimate the correlation degree between lncRNAs and FRGs. The square of correlation coefficient ∣R2∣> 0.3, and *P* < 0.001 was considered as FRlncRNAs. Finally, 1,810 FRlncRNAs were selected.

### 2.3. Identification of the FRlncRNAs Signature

We divided 395 patients into training set (197 cases) and testing set (198 cases) according to a ratio of 1 : 1. The basic characteristics of each group are shown in Table 1. In the training set, univariate Cox regression analysis was used to evaluate the prognostic value of FRlncRNAs. If FRlncRNAs showed *P* < 0.05, they would be included in the least absolute shrinkage and selection operator (lasso) regression. We visualized the coexpression of FRlncRNAs and FRGs using Cytoscape 3.6.0 and the correlation between FRlncRNAs using “igraph package” and “reshape2 package” in R software. Then, the results of lasso regression were included in the multivariate Cox regression model to establish the risk score of each patient. A risk score (∑_*i*=1_^*n*^*β*_*i*_∗(expression of lncRNA_i_)) was established by the expression level of FRlncRNA multiplied by regression coefficient (*β*). Based on the median of risk score, the patients were divided into high-risk group and low-risk group, and the survival rates between the two groups were compared by log-rank test.

In addition, the same formula was used to calculate the risk score of each patient in the testing set and overall set to verify the stability of the established model. The Kaplan-Meier (K-M) curve was conducted to analyze the survival outcomes of each set. The receiver operating characteristic (ROC) curve and its area under the curve (AUC) value were applied to evaluate the specificity and sensitivity of the established model by “ROC package” in R software.

### 2.4. Construction of the Nomogram

An independent prognostic model was established by Cox regression analysis. The 1 -, 3 -, and 5-year survival rates were predicted by establishing nomogram. The stability of the model was evaluated by the index of concordance (C-index), calibration curves, and ROC curves. The basic characteristics of patients were included in the multivariate Cox regression to determine whether the risk score was an independent predictor of prognosis.

### 2.5. Functional Analysis of FRlncRNAs

The functional enrichment of FRlncRNAs was explained by the Gene Set Enrichment Analysis (GSEA) (http://www.broadinstitute.org/gsea/index.jsp). The study accessed the functional enrichment of FRlncRNAs and visualized the Gene Ontology (GO) and the Kyoto Encyclopedia of Genes and Genomes (KEGG) pathways related to ferroptosis. In the enrichment analysis, a two-sided *P* value of <0.05 was considered significant.

### 2.6. Verification of Major FRlncRNAs in External Databases

The Gene Expression Profiling Interactive Analysis (GEPIA, http://gepia.cancer-pku.cn/) database contained RNA-seq and clinical data compiled by TCGA and GTEx databases after standardized transformation. The K-M Plotter database (http://kmplot.com/analysis/) included RNA-seq and prognostic data of 405 BLCA patients. The above two databases were used to verify the relationship between major FRlncRNAs and patient's prognosis.

### 2.7. Statistical Analysis

The K-M analysis was utilized to generate the survival curve in this study, and the log-rank test was used to compare whether there was a difference between the two groups of patients. The Cox regression and lasso regression were applied to access the prognostic effect of FRlncRNAs signature and clinicopathological data. R software (version 3.6) was used for statistical analysis. Statistical tests were two-sided, and studies were considered statistically significant when *P* ≤ 0.05.

## 3. Results

### 3.1. Identification of the FRlncRNAs Signature

**Figure 1:**
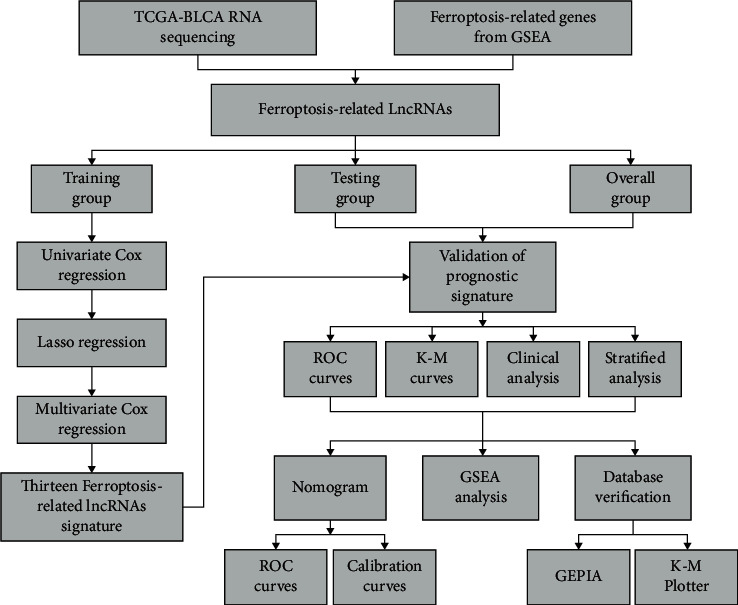
The flowchart of predictive model construction.

**Figure 2:**
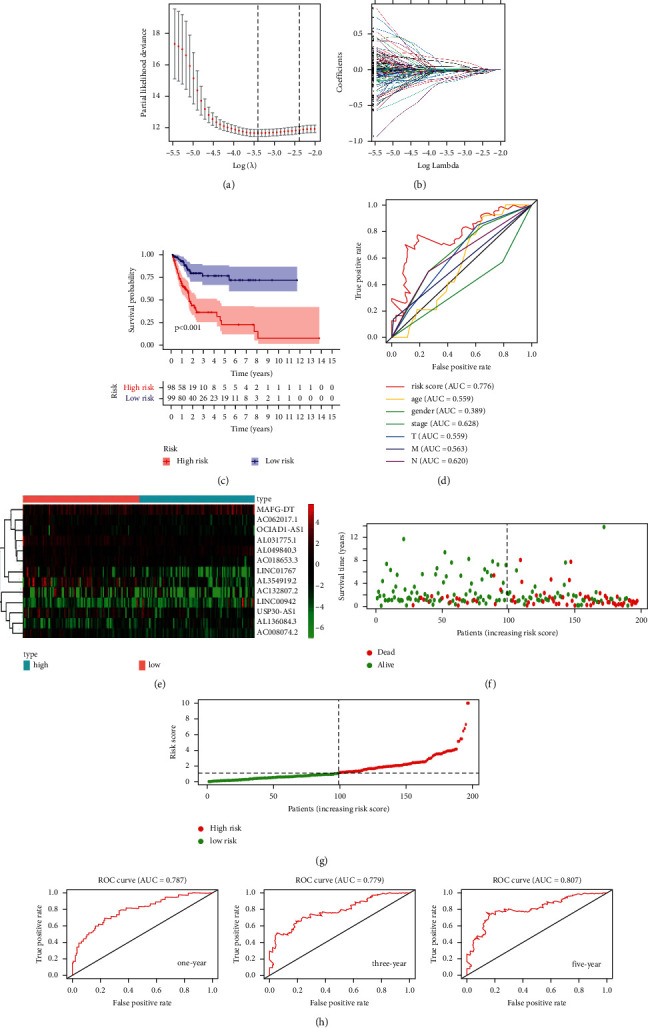
Construction and evaluation of the FRlncRNAs signature in the training set. Lasso coefficient values and vertical dashed lines were calculated at the best log (lambda) value (a), and coefficients (b) of prognostic-related lncRNAs are displayed. (c) The K-M curve showed that the high-risk group had a worse survival rate than the low-risk group (*P* < 0.05). (d) The ROC curve is given for this signature and its AUC value. (e) Heatmap of the 13 FRlncRNAs profiles showed the expression of FRlncRNAs in the high-risk and the low-risk group. (f) Scatter plot showed the correlation between the survival status and risk score of patients. (g) Risk score distribution plot showed the distribution of high-risk and low-risk patients. (h) ROC curves and their AUC value represented 1-, 3-, and 5-year OS.

The flowchart of this research is shown in Figure 1. By univariate Cox regression, we screened a total of 177 FRlncRNAs with prognostic value for patients with BC (*P* < 0.05). Then, we identified 29 FRlncRNAs by lasso regression (Figures 2(a) and 2(b) and Table S1). By multivariate Cox regression, thirteen FRlncRNAs were independent prognostic factors and their coefficients are also shown in Table 2. An FRlncRNAs signature was established based on these 13 FRlncRNAs. The formula of risk score was as follows: risk score = (0.0153*∗*LINC00942) + (0.0550*∗*MAFG-DT) + (0.1824*∗*AL049840.3) + (0.1928 *∗* AL136084.3) – (0.3582*∗*OCIAD1-AS1) – (0.3362*∗*AC062017.1) – (0.2606*∗* AC008074.2) – (0.2419*∗*AC018653.3) – (0.2375*∗* AL031775.1) – (0.1942*∗*USP30-AS1) – (0.1094*∗* LINC01767) – (0.0730*∗* AC132807.2) – (0.0662*∗*AL354919.2).

### 3.2. Prognostic Influence of the FRlncRNAs Signature

**Figure 3:**
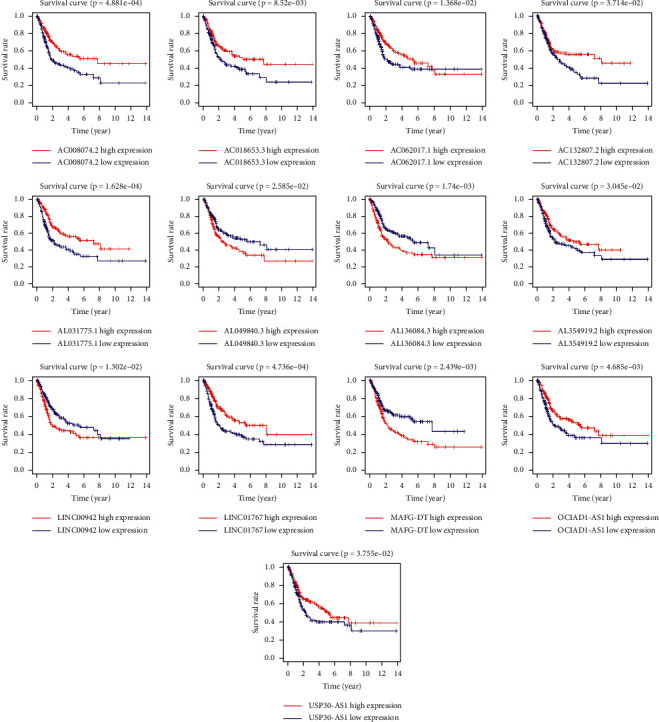
The K-M curve of thirteen prognostic FRlncRNAs. Four FRlncRNAs (LINC00942, MAFG-DT, AL049840.3, and AL136084.3) were independent unfavorable factors. Nine FRlncRNAs (OCIAD1-AS1, AC062017.1, AC008074.2, AC018653.3, AL031775.1, USP30-AS1, LINC01767, AC132807.2, and AL354919.2) were independent beneficial factors.

To access the sensitivity and stability of this signature, the patients in the training set were divided into low-risk group (99 cases) and high-risk group (98 cases) based on the median of risk scores. The K-M curve showed a shorter overall survival (OS) in the high-risk group compared with the low-risk group (*P* < 0.001) (Figure 2(c)). Then, the study used ROC curve to assess the accuracy of the signature and found an AUC value of 0.776 in the training set (Figure 2(d)). The heatmap showed a significant difference in the expression of 13 FRlncRNAs between the high-risk group and the low-risk group (Figure 2(e)). The scatter plot identified that patients with high-risk score had a lower OS than patients with low-risk score (Figure 2(f)). In addition, the distribution map of risk score demonstrated that the high-risk group had a higher risk score than the low-risk group (Figure 2(g)). The AUC value corresponding to 1, 3, and 5 years of OS was 0.787, 0.779, and 0.807 (Figure 2(h)). Besides, we used K-M curves to evaluate the prognostic effect of 13 FRlncRNAs. Among them, four FRlncRNAs (LINC00942, MAFG-DT, AL049840.3, and AL136084.3) were detrimental to the prognosis of patients and nine FRlncRNAs (OCIAD1-AS1, AC062017.1, AC008074.2, AC018653.3, AL031775.1, USP30-AS1, LINC01767, AC132807.2, and AL354919.2) were favorable to the prognosis of patients (Figure 3). In short, this prognostic-related signature had good stability and sensitivity in predicting the OS of patients.

### 3.3. Validation of the FRlncRNAs Signature

**Figure 4:**
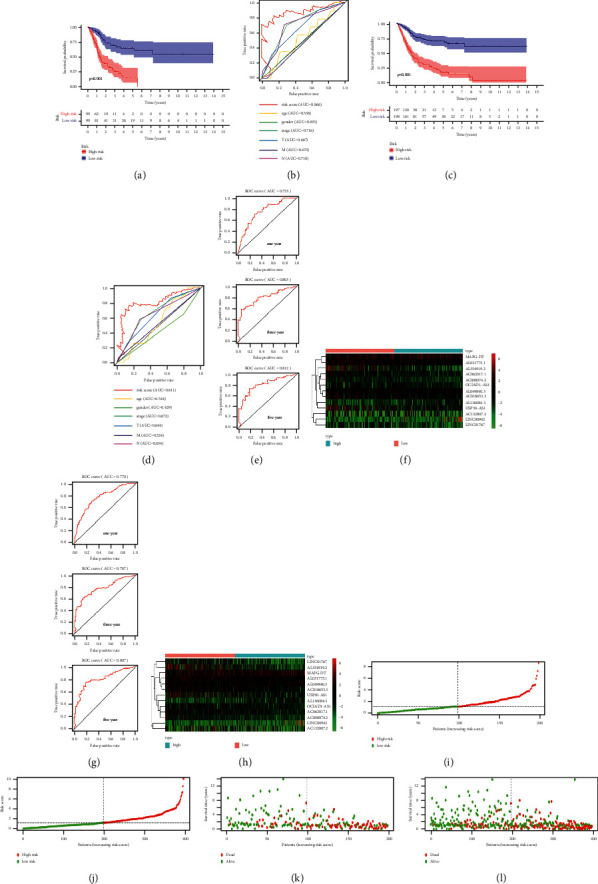
Validation of the FRlncRNAs signature for BC patients in the testing set and overall set. K-M curves showed that the high-risk group had the worse OS than the low-risk group in the testing set (a) and overall set (c). ROC curves and its AUC value in the testing set (b) and overall set (d). ROC curves and their AUC value represented 1-, 3-, and 5-year OS in the testing set (e) and overall set (g). Heatmap of 13 FRlncRNAs profiles showed the expression of FRlncRNAs in the high-risk group and the low-risk group in the testing set (f) and overall set (h). Scatter plot showed the outcomes between the survival status and risk score in the testing set (i) and overall set (j). Risk score distribution plot showed the distribution of high-risk and low-risk BC patients in the testing set (k) and overall set (l).

To evaluate the predictive power of the FRlncRNAs signature, we calculated the risk score of each patient in the testing set and overall set using the same method and divided patients into the low-risk group and the high-risk group. We used the K-M curve to analyze the OS of patients in the testing set (*P* < 0.001) (Figure 4(a)) and overall set (*P* < 0.001) (Figure 4(c)), and the final results were consistent with the training set. Subsequently, the ROC curve showed that this signature has a strong predictive ability in the testing set (AUC = 0.866) (Figure 4(b)) and overall set (AUC = 0.811) (Figure 4(d)) for the OS of patients. Furthermore, ROC time curves and its AUC value also confirmed that this signature had a better prognostic ability for patients in the testing set (1-year AUC = 0.753, 3-year AUC = 0.803, and 5-year AUC = 0.812) (Figure 4(e)) and overall set (1-year AUC = 0.770, 3-year AUC = 0.787, and 5-year AUC = 0.807) (Figure 4(g)). Besides, heatmaps showed that the expression profiles of 13 FRlncRNAs were also consistent with those in the training set (Figures 4(f) and 4(h)). Also, the scatter plot identified that patients with high-risk score had a lower survival rate than patients with low-risk score, and the distribution map of risk score demonstrated that the high-risk group had a higher risk score than the low-risk group (Figures 4(i)–4(l)). These results indicated that the FRlncRNAs signature had a stable prognostic-predictive ability.

### 3.4. Construction and Evaluation of the Prognostic Nomogram

**Figure 5:**
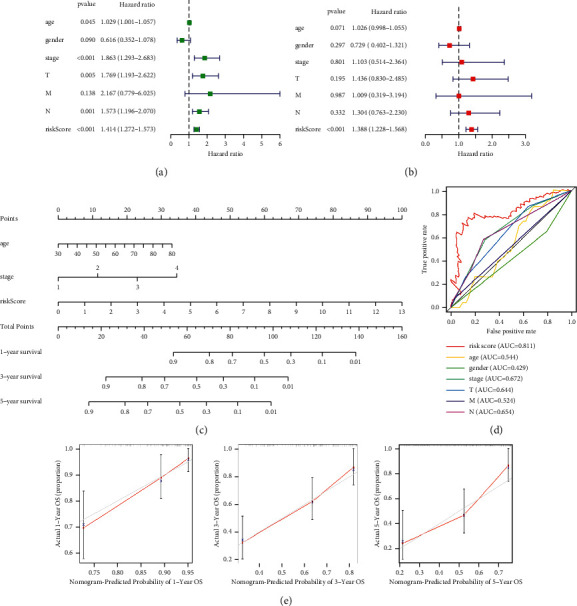
Clinical value of the FRlncRNAs signature in BC patients. The univariate Cox regression showed that risk score and clinicopathological features including age, stage, T stage, and N stage were prognostic-related variables (a). The multivariate Cox regression analysis showed that the risk score was independent prognostic factors (b). Construction of a prognostic nomogram based on risk score and clinicopathological indexes to predict 1-, 3-, and 5-year OS of BC patients (c). The multivariate ROC curve showed that predictive accuracy of risk score was higher than other clinicopathological indexes (d). Calibration curves displayed the concordance between predicted and observed 1-, 3-, and 5-year OS (e).

Based on the result of univariate Cox regression in the overall set, the risk score and stage were independent prognostic indicators in patients with BC, in which the HR of risk score was 1.414 (95% CI: 1.272–1.573, *P* < 0.001, Figure 5(a) and Table 3). The risk score remained an independent prognostic indicator in multivariate Cox regression after controlling for clinical characteristics (HR = 1.388, 95% CI = 1.228–1.568, *P* < 0.001, Figure 5(b)). Subsequently, we included the risk score, patient age, and tumor stage in the nomogram. The nomogram showed that risk score and tumor stage had the greatest contribution to 1 -, 3 -, and 5-year OS in patients with BC (Figure 5(c)). The higher the risk score of samples, the worse the prognosis of patients. The multivariate ROC curve of risk score based on the FRlncRNAs signature and clinicopathologic characteristics indicated that AUC value was 0.811, which was the higher than AUC value of age (0.544), gender (0.429), stage (0.672), T stage (0.644), M stage (0.524), and N stage (0.654), indicating that the predictive power of nomogram was more accurate than that of stage and TNM stage (Figure 5(d)). These results showed that the FRlncRNAs signature can be a good indicator in predicting the prognosis of patients compared with the predictive power of existing lncRNA-related signatures reported in recent studies (Table 4) [17–22]. The C-index of the nomogram was 0.755 (se = 0.032). By AUC of 1-, 3-, and 5-year survival rate, the nomogram had a better prediction for the prognosis of patients with BC (Figure 5(e)).

### 3.5. Stratification Analysis of the Clinicopathological Index

To further evaluate the FRlncRNAs signature and verify its stability in predicting the OS of patients in the high-risk group and the low-risk group, we conducted a stratified analysis based on clinicopathological indexes, including gender (female and male), age (≤65 years and >65 years), stage (I-II and III-IV stages), T stage (T1-2 stage and T3-4 stage), and N stage (N0 stage and N1-3 stages).

**Figure 6:**
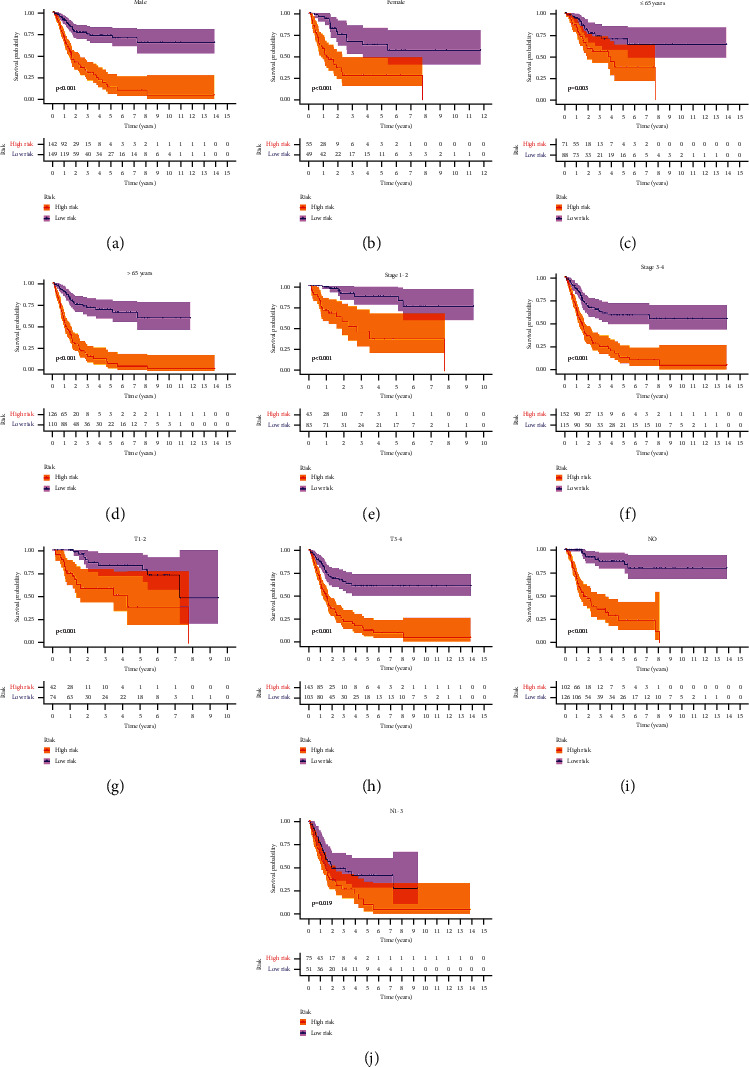
The survival outcomes of the high- and low-risk score subgroup were stratified by clinicopathological indexes. K-M curves showed the survival outcomes of high- and low-risk patients stratified according to gender (male versus female) (a, b), age (≤65 years versus >65 years) (c, d), stage (stages I-II versus stages III-IV) (e, f), T stage (T1-2 versus T3-4) (g, h), and N stage (N0 versus T1-3) (i, j), respectively (all *P* < 0.05).

The result of the K-M curve showed that the OS of the high-risk group was worse than that of the low-risk group in different clinical stratification (*P* < 0.05) (Figures 6(a)–6(j)). Clinical influences of risk score for patients in the training, testing, and overall sets are shown in Table 5.

### 3.6. Gene Functional Analysis

**Figure 7:**
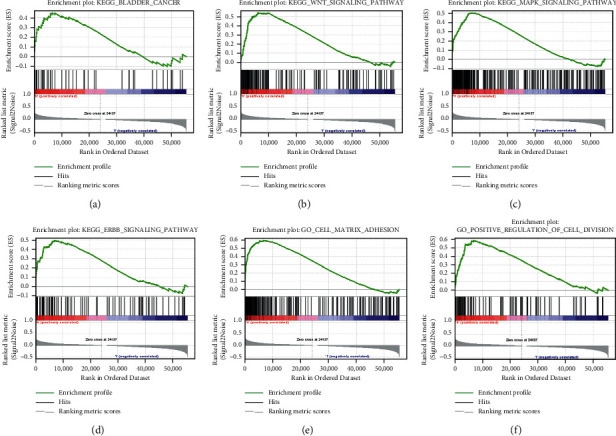
The results of functional analysis based on FRlncRNAs. (a–d) KEGG enrichment analysis; (e, f) GO enrichment analysis.

We totally extracted 936 GO pathways (Table S2) and 51 KEGG pathways (Table S3) from GSEA 4.1.0. KEGG analysis revealed that the signaling pathway such as WNT signaling pathway, mitogen-activated protein kinase (MAPK), and ERBB signaling pathway was significantly enriched in the high-risk group (Figures 7(a)–7(d)). Go analysis found that the functions of FRlncRNAs mainly focused on physiological processes, such as cell-matrix adhesion and positive regulation of cell division (Figures 7(e) and 7(f)).

### 3.7. Construction of the Coexpression Network and Verification of Major FRlncRNAs

**Figure 8:**
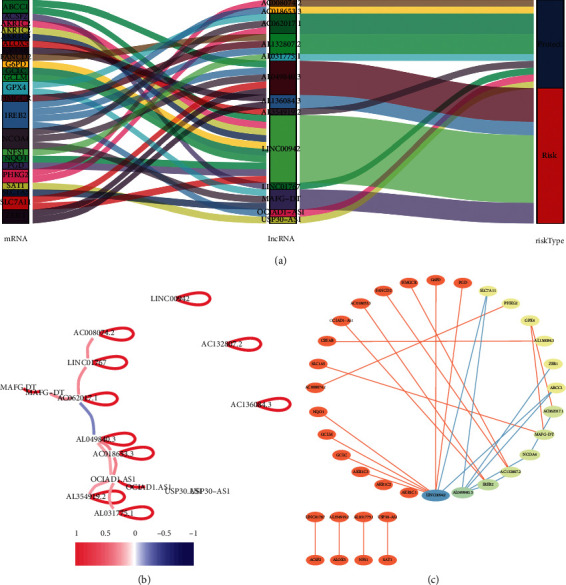
The Sankey diagram and coexpression network of 13 FRlncRNAs and FRGs. (a) Sankey diagram showed the association between FRlncRNAs, FRGs, and risk types. (b) The correlation of 13 FRlncRNAs. (c) The coexpression network between prognostic FRlncRNAs and FRGs in BC.

**Figure 9:**
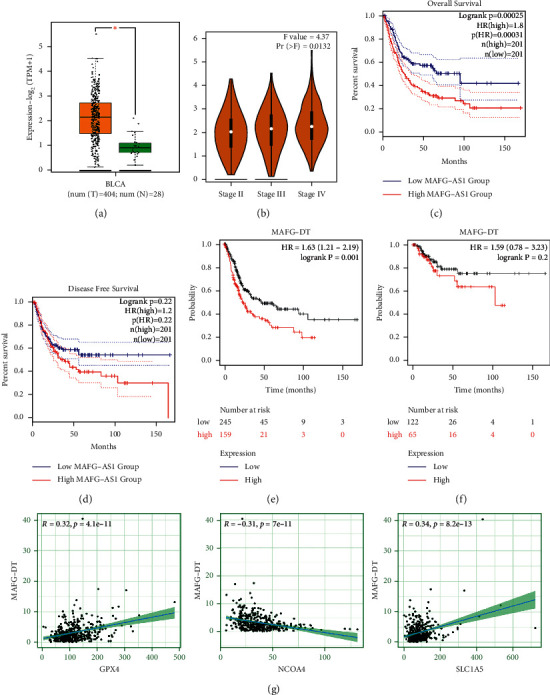
Expression and prognosis of MAFG-DT from the GEPIA and K-M Plotter databases. (a) Expression of MAFG-DT in BC tissues from the GEPIA database (*P* < 0.05). (b) Expression of MAFG-DT based on tumor stage from the GEPIA database (Pr = 0.0132). (c, d) Prognosis of MAFG-DT with the cohort from the GEPIA database. (e) Prognosis of MAFG-DT with the cohort from the K-M Plotter database. (g) Linear correlation analysis of MAFG-DT and protein-coding genes (GPX4, NCOA4, and SLC1A5).

The Sankey diagram showed the association between FRlncRNAs, FRGs, and risk types (Figure 8(a)). The correlation of 13 FRlncRNAs is shown in Figure 8(b). The coexpression network between prognostic FRlncRNAs and FRGs is shown in Figure 8(c). After literature search, we selected MAFG-DT (MAFG-AS1) for further study. We verified the expression levels and survival outcomes of MAFG-DT in two external databases (GEPIA and K-M Plotter). In the cohort from the GEPIA database, the expression of MAFG-DT in tumor tissues was higher than that in normal tissues (Figure 9(a)), and the expression level of MAFG-DT differed across the three pathological stages, which may indicate that MAFG-DT was closely related to the prognosis of patients (Figure 9(b)). As shown in the GEPIA database, high MAFG-DT expression levels were associated with poor prognosis (*P* < 0.05) (Figures 9(c) and 9(d)). In the 405 patients from the K-M Plotter database, 5-year OS in the group with high expression of MAFG-DT was lower than that in the group with low expression (*P* < 0.05) (Figures 9(e) and 9(f)). In order to intuitively show the expression correlation of MAFG-DT and FRGs, we made a linear correlation graph including GPX4, NCOA4, and SLC1A5 (Figure 9(g)). In conclusion, these results showed that the expression of MAFG-DT was higher in BC tissues, but with the increase of the stage, the expression of MAFG-DT also increased, and there was a significant correlation with survival outcomes.

## 4. Discussion

Despite advances in surgery and chemotherapy, the prognosis of patients with advanced and metastatic BC remained unsatisfactory [23]. Furthermore, because of the different causative molecules, although some patients have the same TNM stage or similar risk factors, they may have different clinical outcomes. Therefore, molecular biomarkers to predict tumor prognosis are of great importance [24, 25]. Ferroptosis may be associated with biological behaviors such as proliferation, invasion, and metastasis in BC [6, 7]. And lncRNAs may play key regulatory roles in ferroptosis-related biological processes of malignant tumor cells [26, 27]. However, a prognostic tool based on FRlncRNAs for patients with BC is still lacking.

In our study, FRlncRNAs were collected by establishing a coexpression network of lncRNAs and FRGs. Initially, we identified 29 FRlncRNAs related to prognosis and constructed a prognostic model comprising 13 FRlncRNAs (LINC00942, MAFG-DT, AL049840.3, AL136084.3, OCIAD1-AS1, AC062017.1, AC008074.2, AC018653.3, AL031775.1, USP30-AS1, LINC01767, AC132807.2, and AL354919.2) via multivariate Cox regression and lasso regression. K-M curve showed that the OS of patients with high-risk score were shorter than those of patients with low-risk score. In addition, the ROC curve proved that the 13 FRlncRNAs signature was highly sensitive and specific prognostic markers in BC. Besides, this result was further verified in the testing set and overall set. The 13 FRlncRNAs' signature was also associated with poor OS of BC patients in different subgroups, especially in age, gender, AJCC stage, T stage, and N stage. Next, a nomogram was used to count the risk score and can predict the OS of patients. The calibration curve showed that the signature had a higher sensitivity and clinical applicability than the traditional standard. Then, we established an FRlncRNA-mRNAs coexpression network to analyze the effect of the 13 FRlncRNAs' signature. And we also analyzed the correlation between 13 FRlncRNAs. The GSEA analysis revealed that signaling pathways such as WNT, MAPK, ERBB, cell-matrix adhesion, and positive regulation of cell division were significantly enriched in the high-risk group. Finally, we verified the major FRlncRNAs in two external databases. The results showed that the expression of MAFG-DT was higher in BC tissues, but with the increase of the stage, the expression of MAFG-DT also increased, and there was a significant correlation with survival outcomes. Correlation analysis found that MAFG-DT and protein-coding genes (GPX4, NCOA4, and SLC1A5) were obviously related.

Currently, multiple FRlncRNAs have been reported to be associated with poor prognosis in a variety of tumors. Among them, LINC00942 exerts its functions as an oncogene in promoting METTL14-mediated m6A methylation and regulating the expression and stability of its target genes CXCR4 and CYP1B1 in breast cancer (BRCA) initiation and progression, which provides new targets and crosstalk m6A epigenetic modification mechanism for BRCA prevention and treatment [28]. Six FRlncRNAs were included in the relevant clinical prediction models, which showed a good prediction effect for patients with BC. Tong et al. found that AL049840.3, AL031775.1, and USP30-AS1 may predict the prognosis and progression of patients with BC as an epithelial-mesenchymal transition- (EMT-) related lncRNAs [29]. Wang et al. found that OCIAD1-AS1 and AL354919.2 may predict the prognosis and progression of patients with BC as immune-related lncRNAs [30]. USP30-AS1 has also been found to be associated with autophagy, which may be involved in the diagnosis and prognosis of BC [31].

As a major FRlncRNA, MAFG-DT is a tumorigenic lncRNA in a variety of cancers. Firstly, MAFG-DT as a part of lncRNAs signature of tumor-infiltrating B lymphocytes had some predictive value for the survival outcomes and immunotherapy response of patients with antiprogrammed death-1 (PD-1) therapy and added significant predictive power to current immune checkpoint gene markers [32]. Next, Li et al. reported that silencing of MAFG-AS1 inhibited BC cell proliferation, metastasis, and invasion, while overexpression of MAFG-AS1 in BC cell had opposite biological effects, and it was further confirmed that MAFG-AS1/miR-143-3p/COX-2 axis was involved in the progression of BC [33]. Besides, Xiao et al. showed that MAFG-AS1 can promote BC proliferation, invasion, metastasis, and EMT in vitro and in vivo. Mechanistically, MAFG-AS1 directly binding to Hu antigen R (HuR) could recruit ubiquitin-specific proteinase 5 (USP5) to prevent HuR from degrading by ubiquitination [34]. For six remaining FRlncRNAs (AL136084.3, AC062017.1, AC008074.2, AC018653.3, LINC01767, and AC132807.2), there were no studies to report their prognostic roles in cancer. Thus, more researches were necessary to explore how these lncRNAs affect the prognosis of patients with BC through ferroptosis exactly.

Currently, multiple studies have highlighted the role of lncRNAs in the pathogenesis of BC. These lncRNAs were downregulated or upregulated during the origin, proliferation, and migration of BC. Therefore, it is very necessary to clarify the physiological roles of lncRNAs and their contributions to the development and progression of BC. For example, lncRNAs can contribute to mRNA degradation and failure of protein translation by mediating posttranscriptional gene silencing [35]. In addition, lncRNA can also remodel chromatin structure by directing the formation of heterochromatin, thereby affecting the apparent phenotype of cells [36]. LncRNAs can also be involved in both cis- and transgene regulation, thereby enhancing or repressing gene expression [35]. These effects are crucial for the proliferation, differentiation, regeneration, and apoptosis of human cells, thereby helping to maintain the balance of the normal physiological function of the body [37, 38]. However, relative to the wide application of lncRNAs in BC diagnosis, researches of lncRNA-targeting therapies are relatively scarce. Since lncRNAs diagnostic is noninvasive and can detect the occurrence of BC more rapidly compared with cystoscopy, a commonly used diagnostic tool [39].

Ferroptosis can synergistically enhance antitumor activity in combination with immune checkpoint inhibitors (ICIs), even in ICI-resistant types [40]. Currently, there are fewer studies analyzing the relationship between ICIs and ferroptosis. Accumulating evidence has found that microRNAs (miRNAs) and lncRNA were crucial in mediating the regulation of ferroptosis. Nrf2 reduces ROS generation by inhibiting iron absorption [41]. As such, miRNAs can disrupt the process of ferroptosis by regulating the expression of Nrf2 [42, 43]. Meanwhile, miRNAs were involved in regulating iron absorption, transport, storage, and metabolism. In recent years, multiple regulators of ferroptosis have been discovered, including ATF3/4, SLC7A11, and ACSl4. Importantly, lncRNAs play a key role in regulating the expression of these factors [44].

Ferroptosis, as a new form of cell death, may provide a new direction for tumor therapy. However, many key questions remain unresolved, such as how ferroptosis intersects with other cell deaths and host immunogenicity is less well studied. Therefore, this study explored ferroptosis-related biomarkers that can be used to predict BC prognosis, which may provide references for therapeutic modalities of cancer. However, the current study has some weaknesses. First, the data source of this analysis was single, and the amount of data included was small, so the results may have some bias. Second, the study was retrospective, and prospective studies may be needed to demonstrate the prognostic function of FRlncRNAs. Third, to further validate the stability and accuracy of this prognostic model, our established prognostic model needs further analysis in other independent cohorts. Fourth, relevant functional experiments should be performed to further analyze the underlying molecular mechanisms by which FRlncRNAs affect the development and progression of BC.

## 5. Conclusions

The coexpression network of FRlncRNA-mRNA provided a valuable source for revealing the function of FRlncRNAs in BC. Thirteen FRlncRNAs were considered to be significantly associated with OS of patients with BC. An FRlncRNAs signature that was composed of thirteen FRlncRNAs was used to differentiate patients at different risks, and it was a significantly independent factor for patients with BC. Therefore, the thirteen FRlncRNAs and their signature might be molecular biomarkers and therapeutic targets for patients with BC.

## Figures and Tables

**Table 1:** Characteristics of BC patients included in this study.

Variable		Overall set (*n* = 395)	Training set (*n* = 197)	Testing set (*n* = 198)
Age (mean ± SD, years)		67.82 ± 10.52	67.11 ± 10.36	68.53 ± 10.63
Gender (*n*)	Male	291	149	142
	Female	104	48	56
Stage (*n*)	Stages I-II	126	71	55
	Stages III-IV	267	126	141
	UA	2	0	2
T stage (*n*)	T0	1	0	1
	T1-2	116	62	54
	T3-4	246	119	127
	UA	32	16	16
N stage (*n*)	N0	228	116	112
	N1-3	126	56	70
	UA	41	25	16
M stage (*n*)	M0	189	105	84
	M1	10	4	6
	UA	196	88	108
Survival time (mean ± SD, years)		2.14 ± 2.23	2.12 ± 2.23	2.15 ± 2.23
Risk scores (mean ± SD)		1.61 ± 1.63	1.65 ± 1.86	1.56 ± 1.35

BC, bladder cancer; SD, standard deviation.

**Table 2:** The HRs, *P* values, and coefficients of 13 ferroptosis-related lncRNAs in the multivariate Cox regression analysis.

lncRNA	Coefficient	HR	95% CI of HR	*P* value
AL136084.3	0.1928	1.21	1.01–1.45	0.034
USP30-AS1	−0.1942	0.82	0.74–0.91	<0.001
AC062017.1	−0.3362	0.71	0.51–1.00	0.052
OCIAD1-AS1	−0.3582	0.70	0.50–0.99	0.042
LINC00942	0.0153	1.02	1.00–1.03	0.017
LINC01767	−0.1094	0.90	0.76–1.06	0.189
AL049840.3	0.1824	1.20	1.00–1.43	0.045
AL354919.2	−0.0662	0.94	0.87–1.01	0.098
MAFG-DT	0.0550	1.06	1.02–1.10	0.003
AC008074.2	−0.2606	0.77	0.55–1.08	0.132
AL031775.1	−0.2375	0.79	0.64–0.97	0.023
AC018653.3	−0.2419	0.79	0.63–0.98	0.034
AC132807.2	−0.0730	0.93	0.85–1.02	0.133

HR, hazard rate; CI, confidence interval.

**Table 3:** Univariate and multivariate Cox regression analysis of the association between clinicopathological indexes (including risk score) and overall survival of BC patients in the training, testing, and overall sets.

Variable	Overall set				Training set				Testing set			
	Univariate		Multivariate		Univariate		Multivariate		Univariate		Multivariate	
	HR (95% CI)	*P*	HR (95% CI)	*P*	HR (95% CI)	*P*	HR (95% CI)	*P*	HR (95% CI)	*P*	HR (95% CI)	*P*
Age	1.029 (1.001–1.057)	0.045	1.026 (0.998–1.055)	0.071	1.008 (0.971–1.047)	0.674	1.021 (0.976–1.068)	0.365	1.046 (1.003–1.090)	0.035	1.011 (0.965–1.059)	0.652
Gender	0.616 (0.352–1.078)	0.090	0.729 (0.402–1.321)	0.297	0.623 (0.299–1.296)	0.206	0.745 (0.340–1.635)	0.463	0.687 (0.287–1.648)	0.401	1.070 (0.415–2.761)	0.888
Stage	1.863 (1.293–2.683)	<0.001	1.103 (0.514–2.364)	0.801	1.869 (1.157–3.018)	0.011	1.297 (0.478–3.519)	0.610	1.864 (1.070–3.250)	0.028	0.541 (0.162–1.808)	0.318
T stage	1.769 (1.193–2.622)	0.005	1.436 (0.830–2.485)	0.195	1.410 (0.862–2.308)	0.171	1.019 (0.509–2.041)	0.958	2.499 (1.300–4.804)	0.006	3.514 (1.319–9.358)	0.012
N stage	1.573 (1.196–2.070)	0.001	1.304 (0.763–2.230)	0.332	1.656 (1.103–2.488)	0.015	1.281 (0.574–2.860)	0.545	1.551 (1.058–2.275)	0.025	2.008 (0.902–4.472)	0.088
M stage	2.167 (0.779–6.025)	0.138	1.009 (0.319–3.194)	0.987	4.857 (1.108–21.287)	0.036	1.783 (0.372–8.553)	0.470	1.392 (0.326–5.949)	0.655	0.322 (0.053–1.946)	0.217
Risk score	1.414 (1.272–1.573)	<0.001	1.388 (1.228–1.568)	<0.001	1.350 (1.190–1.532)	<0.001	1.350 (1.156–1.578)	<0.001	1.507 (1.193–1.904)	<0.001	1.446 (1.088–1.924)	0.011

BC, bladder cancer; HR, hazard ratio; CI, confidence interval.

**Table 4:** The comparison of studies about existing lncRNAs signatures for bladder cancer.

References	Signature	Database	Gene list	Survival event	AUC value
Wu et al. [17]	Mutation-derived genomic instability-associated lncRNA	TCGA	CFAP58-DT, MIR100HG, LINC02446, AC078880.3, and LINC01833	OS	0.756
Zhao et al. [18]	Immune-related lncRNA	TCGA	AC005674.2, AC090948.1, TFAP2A-AS1, AL354919.2, AC011468.1, and AC018809.2	OS	0.77
Luo et al. [19]	Immune-related LncRNA	TCGA, GEO	RP11-89, PSORS1C3, LINC02672, and MIR100HG	OS	0.642
Du et al. [20]	Stromal EMT-related LncRNA	TCGA	AL583785.1, TMEM51-AS1, AC073534.1, LINC01711, and LINC02446	OS	0.799
Qing et al. [21]	Extracellular matrix-related lncRNA	TCGA	SNHG12, MAFG- DT, ASMTL-AS1, LINC02321, LINC01322, and LINC00922	OS	0.686
Sun et al. [22]	Autophagy-related lncRNA	TCGA	LINC02178, AC108449.2, Z83843.1, FAM13A-AS1, and USP30-AS1	OS	0.710
Our study	Ferroptosis-related lncRNA	TCGA	LINC00942, MAFG-DT, AL049840.3, AL136084.3, OCIAD1-AS1, AC062017.1, AC008074.2, AC018653.3, AL031775.1, USP30-AS1, LINC01767, AC132807.2, and AL354919.2	OS	0.811

LncRNA, long noncoding RNA; EMT, epithelial to mesenchymal transition; TCGA, the Cancer Genome Atlas; AUC, area under the curve; OS, overall survival.

**Table 5:** Clinical influences of risk score signature for BC patients in the training, testing, and overall sets.

Variable		Risk score signature											
		Overall set				Training set				Testing set			
		*n*	Mean ± SD	*t*	*P*	*n*	Mean ± SD	*t*	*P*	*n*	Mean ± SD	*t*	*P*
Age	≤65	62	1.477 ± 1.867	0.490	0.625	34	1.940 ± 2.412	1.029	0.309	28	1.050 ± 0.970	−2.253	0.027
	>65	101	1.609 ± 1.425			52	1.470 ± 1.397			49	1.994 ± 2.381		
Gender	Female	37	2.133 ± 2.414	1.815	0.077	21	2.167 ± 2.902	1.032	0.313	16	2.088 ± 1.658	1.861	0.078
	Male	126	1.384 ± 1.263			65	1.490 ± 1.372			61	1.270 ± 1.136		
Stage	I-II	48	1.017 ± 0.964	−3.512	0.001	27	1.047 ± 1.110	−2.578	0.012	21	0.978 ± 0.762	−2.528	0.014
	III-IV	115	1.778 ± 1.781			59	1.934 ± 2.074			56	1.613 ± 1.410		
T stage	T1-2	53	1.075 ± 0.966	−3.253	0.001	30	1.100 ± 1.086	−2.465	0.016	23	1.043 ± 0.807	−2.208	0.031
	T3-4	110	1.784 ± 1.814			56	1.953 ± 2.122			54	1.609 ± 1.425		
N stage	N0	112	1.444 ± 1.750	−1.447	0.150	60	1.582 ± 2.078	−0.661	0.511	52	1.284 ± 1.275	−1.534	0.132
	N1-3	51	1.795 ± 1.270			26	1.824 ± 1.270			25	1.765 ± 1.295		
M stage	M0	156	1.506 ± 1.621	−2.214	0.063	83	1.579 ± 1.854	−8.767	0.000	73	1.424 ± 1.314	−0.651	0.553
	M1	7	2.608 ± 1.271			3	3.775 ± 0.253			4	1.733 ± 0.898		

SD, standard deviation.

## Data Availability

The datasets used to support the findings of this study are available from the TCGA (https://portal.gdc.cancer.gov/repository) database.

## Supplementary Materials

Supplementary Materials Supplementary Table S1: twenty-nine FRlncRNAs identified after univariate Cox regression and lasso regression. Supplementary Table S2: gene set enrichment GO analysis results according to the signature of thirteen FRlncRNAs (top 20 *P* values). Supplementary Table S3: gene set enrichment KEGG analysis results based on the signature of thirteen FRlncRNAs (top 20 *P* values). .
