# Supplementary material for: Identification of a Nomogram from Ferroptosis-Related Long Noncoding RNAs Signature to Analyze Overall Survival in Patients with Bladder Cancer
Source: J Oncol. 2021 Aug 26;2021:8533464. doi: 10.1155/2021/8533464 (PMC8413054; doi:10.1155/2021/8533464)
Supplement: Supplementary Materials — Supplementary Table S1: twenty-nine FRlncRNAs identified after univariate Cox regression and lasso regression. Supplementary Table S2: gene set enrichment GO analysis results according to the signature of thirteen FRlncRNAs (top 20 P values). Supplementary Table S3: gene set enrichment KEGG analysis results based on the signature of thirteen FRlncRNAs (top 20 P values). . [file 8533464.f1.docx]

Table S1. 29 FRlncRNAs identified by lasso regression analysis.

| AL136084.3 |
| --- |
| USP30-AS1 |
| AC104564.3 |
| AC022150.2 |
| LINC01560 |
| AC132807.2 |
| AC018653.3 |
| AL031775.1 |
| AC008074.2 |
| AC009812.1 |
| MAFG-DT |
| TTC28-AS1 |
| PACERR |
| THUMPD3-AS1 |
| AL354919.2 |
| STAG3L5P-PVRIG2P-PILRB |
| AL049840.3 |
| AC008035.1 |
| AC104532.2 |
| AC078778.1 |
| LINC01767 |
| LINC00942 |
| SNHG18 |
| OCIAD1-AS1 |
| AC009283.1 |
| AJ271736.1 |
| AC024060.1 |
| AC099518.2 |
| AC062017.1 |

Table S2 Gene set enrichment GO analysis results according to the signature of thirteen ferroptosis-related lncRNAs (Top 20 p-value).

| Name | SIZE | ES | NES | NOM p-value | FDR q-value | FWER p-value | RANK AT MAX | LEADING EDGE |
| --- | --- | --- | --- | --- | --- | --- | --- | --- |
| [GO_ATP_SYNTHESIS_COUPLED_ELECTRON_TRANSPORT](http://www.gsea-msigdb.org/gsea/msigdb/cards/GO_ATP_SYNTHESIS_COUPLED_ELECTRON_TRANSPORT) | 98 | -0.69 | -2.08 | 0.008 | 0.393 | 0.241 | 7159 | tags=56%, list=13%, signal=64% |
| [GO_RESPIRASOME](http://www.gsea-msigdb.org/gsea/msigdb/cards/GO_RESPIRASOME) | 101 | -0.69 | -2.07 | 0.004 | 0.215 | 0.257 | 9390 | tags=58%, list=17%, signal=70% |
| [GO_RESPIRATORY_CHAIN_COMPLEX](http://www.gsea-msigdb.org/gsea/msigdb/cards/GO_RESPIRATORY_CHAIN_COMPLEX) | 85 | -0.71 | -2.06 | 0.004 | 0.161 | 0.270 | 9390 | tags=64%, list=17%, signal=76% |
| [GO_MITOCHONDRIAL_ELECTRON_TRANSPORT_NADH_TO_UBIQUINONE](http://www.gsea-msigdb.org/gsea/msigdb/cards/GO_MITOCHONDRIAL_ELECTRON_TRANSPORT_NADH_TO_UBIQUINONE) | 55 | -0.72 | -2.06 | 0.002 | 0.125 | 0.274 | 7159 | tags=64%, list=13%, signal=73% |
| [GO_RESPIRATORY_ELECTRON_TRANSPORT_CHAIN](http://www.gsea-msigdb.org/gsea/msigdb/cards/GO_RESPIRATORY_ELECTRON_TRANSPORT_CHAIN) | 116 | -0.64 | -2.05 | 0.008 | 0.119 | 0.304 | 10736 | tags=58%, list=19%, signal=72% |
| [GO_OXIDOREDUCTASE_ACTIVITY_ACTING_ON_NAD_P_H_QUINONE_OR_SIMILAR_COMPOUND_AS_ACCEPTOR](http://www.gsea-msigdb.org/gsea/msigdb/cards/GO_OXIDOREDUCTASE_ACTIVITY_ACTING_ON_NAD_P_H_QUINONE_OR_SIMILAR_COMPOUND_AS_ACCEPTOR) | 61 | -0.69 | -2.03 | 0.010 | 0.120 | 0.341 | 8587 | tags=56%, list=16%, signal=66% |
| [GO_MONOCARBOXYLIC_ACID_CATABOLIC_PROCESS](http://www.gsea-msigdb.org/gsea/msigdb/cards/GO_MONOCARBOXYLIC_ACID_CATABOLIC_PROCESS) | 137 | -0.55 | -2.03 | 0.002 | 0.105 | 0.343 | 10337 | tags=47%, list=19%, signal=58% |
| [GO_MITOCHONDRIAL_RESPIRATORY_CHAIN_COMPLEX_ASSEMBLY](http://www.gsea-msigdb.org/gsea/msigdb/cards/GO_MITOCHONDRIAL_RESPIRATORY_CHAIN_COMPLEX_ASSEMBLY) | 101 | -0.66 | -2.02 | 0.012 | 0.097 | 0.353 | 13681 | tags=71%, list=25%, signal=95% |
| [GO_OXIDATIVE_PHOSPHORYLATION](http://www.gsea-msigdb.org/gsea/msigdb/cards/GO_OXIDATIVE_PHOSPHORYLATION) | 146 | -0.62 | -2.00 | 0.008 | 0.108 | 0.406 | 7159 | tags=49%, list=13%, signal=57% |
| [GO_OXIDOREDUCTASE_COMPLEX](http://www.gsea-msigdb.org/gsea/msigdb/cards/GO_OXIDOREDUCTASE_COMPLEX) | 110 | -0.59 | -1.99 | 0.006 | 0.108 | 0.425 | 8684 | tags=48%, list=16%, signal=57% |
| [GO_FATTY_ACID_CATABOLIC_PROCESS](http://www.gsea-msigdb.org/gsea/msigdb/cards/GO_FATTY_ACID_CATABOLIC_PROCESS) | 113 | -0.54 | -1.99 | 0.002 | 0.107 | 0.442 | 10337 | tags=47%, list=19%, signal=58% |
| [GO_U1_SNRNP](http://www.gsea-msigdb.org/gsea/msigdb/cards/GO_U1_SNRNP) | 34 | -0.70 | -1.97 | 0.000 | 0.112 | 0.473 | 7452 | tags=50%, list=13%, signal=58% |
| [GO_FATTY_ACID_BETA_OXIDATION](http://www.gsea-msigdb.org/gsea/msigdb/cards/GO_FATTY_ACID_BETA_OXIDATION) | 74 | -0.56 | -1.97 | 0.006 | 0.109 | 0.484 | 7539 | tags=45%, list=14%, signal=52% |
| [GO_CYTOCHROME_COMPLEX](http://www.gsea-msigdb.org/gsea/msigdb/cards/GO_CYTOCHROME_COMPLEX) | 34 | -0.71 | -1.96 | 0.008 | 0.115 | 0.512 | 9317 | tags=62%, list=17%, signal=74% |
| [GO_NADH_DEHYDROGENASE_COMPLEX](http://www.gsea-msigdb.org/gsea/msigdb/cards/GO_NADH_DEHYDROGENASE_COMPLEX) | 50 | -0.72 | -1.95 | 0.008 | 0.118 | 0.531 | 8587 | tags=66%, list=16%, signal=78% |
| [GO_NADH_DEHYDROGENASE_COMPLEX_ASSEMBLY](http://www.gsea-msigdb.org/gsea/msigdb/cards/GO_NADH_DEHYDROGENASE_COMPLEX_ASSEMBLY) | 65 | -0.68 | -1.95 | 0.012 | 0.111 | 0.531 | 7589 | tags=55%, list=14%, signal=64% |
| [GO_ATP_SYNTHESIS_COUPLED_PROTON_TRANSPORT](http://www.gsea-msigdb.org/gsea/msigdb/cards/GO_ATP_SYNTHESIS_COUPLED_PROTON_TRANSPORT) | 24 | -0.78 | -1.94 | 0.006 | 0.110 | 0.539 | 5199 | tags=63%, list=9%, signal=69% |
| [GO_NADH_DEHYDROGENASE_ACTIVITY](http://www.gsea-msigdb.org/gsea/msigdb/cards/GO_NADH_DEHYDROGENASE_ACTIVITY) | 46 | -0.72 | -1.94 | 0.012 | 0.107 | 0.542 | 8587 | tags=65%, list=16%, signal=77% |
| [GO_CYTOCHROME_COMPLEX_ASSEMBLY](http://www.gsea-msigdb.org/gsea/msigdb/cards/GO_CYTOCHROME_COMPLEX_ASSEMBLY) | 36 | -0.65 | -1.93 | 0.017 | 0.108 | 0.559 | 9481 | tags=56%, list=17%, signal=67% |
| [GO_OXIDOREDUCTASE_ACTIVITY_ACTING_ON_A_HEME_GROUP_OF_DONORS](http://www.gsea-msigdb.org/gsea/msigdb/cards/GO_OXIDOREDUCTASE_ACTIVITY_ACTING_ON_A_HEME_GROUP_OF_DONORS) | 29 | -0.70 | -1.93 | 0.004 | 0.104 | 0.561 | 6813 | tags=55%, list=12%, signal=63% |

Table S3 Gene set enrichment KEGG analysis results based on the signature of thirteen ferroptosis-related lncRNAs (Top 20 p-value)

| Name | SIZE | ES | NES | NOM p-value | FDR q-value | FWER p-value | RANK AT MAX | LEADING EDGE |
| --- | --- | --- | --- | --- | --- | --- | --- | --- |
| [KEGG_FOCAL_ADHESION](http://www.gsea-msigdb.org/gsea/msigdb/cards/KEGG_FOCAL_ADHESION) | 199 | 0.69 | 2.50 | 0.000 | 0.000 | 0.000 | 6319 | tags=59%, list=11%, signal=67% |
| [KEGG_REGULATION_OF_ACTIN_CYTOSKELETON](http://www.gsea-msigdb.org/gsea/msigdb/cards/KEGG_REGULATION_OF_ACTIN_CYTOSKELETON) | 213 | 0.61 | 2.48 | 0.000 | 0.000 | 0.000 | 6172 | tags=49%, list=11%, signal=55% |
| [KEGG_ARRHYTHMOGENIC_RIGHT_VENTRICULAR_CARDIOMYOPATHY_ARVC](http://www.gsea-msigdb.org/gsea/msigdb/cards/KEGG_ARRHYTHMOGENIC_RIGHT_VENTRICULAR_CARDIOMYOPATHY_ARVC) | 74 | 0.73 | 2.42 | 0.000 | 0.000 | 0.000 | 6296 | tags=65%, list=11%, signal=73% |
| [KEGG_HYPERTROPHIC_CARDIOMYOPATHY_HCM](http://www.gsea-msigdb.org/gsea/msigdb/cards/KEGG_HYPERTROPHIC_CARDIOMYOPATHY_HCM) | 83 | 0.71 | 2.41 | 0.000 | 0.000 | 0.000 | 6172 | tags=60%, list=11%, signal=68% |
| [KEGG_DILATED_CARDIOMYOPATHY](http://www.gsea-msigdb.org/gsea/msigdb/cards/KEGG_DILATED_CARDIOMYOPATHY) | 90 | 0.69 | 2.38 | 0.000 | 0.000 | 0.000 | 6428 | tags=57%, list=12%, signal=64% |
| [KEGG_GAP_JUNCTION](http://www.gsea-msigdb.org/gsea/msigdb/cards/KEGG_GAP_JUNCTION) | 90 | 0.63 | 2.36 | 0.000 | 0.000 | 0.000 | 8709 | tags=60%, list=16%, signal=71% |
| [KEGG_ADHERENS_JUNCTION](http://www.gsea-msigdb.org/gsea/msigdb/cards/KEGG_ADHERENS_JUNCTION) | 73 | 0.63 | 2.31 | 0.000 | 0.000 | 0.000 | 3786 | tags=49%, list=7%, signal=53% |
| [KEGG_MELANOMA](http://www.gsea-msigdb.org/gsea/msigdb/cards/KEGG_MELANOMA) | 71 | 0.62 | 2.31 | 0.000 | 0.000 | 0.000 | 7543 | tags=55%, list=14%, signal=64% |
| [KEGG_ECM_RECEPTOR_INTERACTION](http://www.gsea-msigdb.org/gsea/msigdb/cards/KEGG_ECM_RECEPTOR_INTERACTION) | 84 | 0.74 | 2.28 | 0.000 | 0.000 | 0.001 | 8518 | tags=74%, list=15%, signal=87% |
| [KEGG_AXON_GUIDANCE](http://www.gsea-msigdb.org/gsea/msigdb/cards/KEGG_AXON_GUIDANCE) | 129 | 0.57 | 2.28 | 0.000 | 0.000 | 0.001 | 7600 | tags=52%, list=14%, signal=60% |
| [KEGG_PATHWAYS_IN_CANCER](http://www.gsea-msigdb.org/gsea/msigdb/cards/KEGG_PATHWAYS_IN_CANCER) | 325 | 0.53 | 2.27 | 0.000 | 0.000 | 0.001 | 8119 | tags=48%, list=15%, signal=56% |
| [KEGG_MELANOGENESIS](http://www.gsea-msigdb.org/gsea/msigdb/cards/KEGG_MELANOGENESIS) | 101 | 0.55 | 2.23 | 0.000 | 0.000 | 0.002 | 9935 | tags=51%, list=18%, signal=63% |
| [KEGG_WNT_SIGNALING_PATHWAY](http://www.gsea-msigdb.org/gsea/msigdb/cards/KEGG_WNT_SIGNALING_PATHWAY) | 151 | 0.54 | 2.20 | 0.000 | 0.000 | 0.003 | 6540 | tags=47%, list=12%, signal=53% |
| [KEGG_MAPK_SIGNALING_PATHWAY](http://www.gsea-msigdb.org/gsea/msigdb/cards/KEGG_MAPK_SIGNALING_PATHWAY) | 267 | 0.50 | 2.18 | 0.000 | 0.000 | 0.004 | 7205 | tags=45%, list=13%, signal=51% |
| [KEGG_TIGHT_JUNCTION](http://www.gsea-msigdb.org/gsea/msigdb/cards/KEGG_TIGHT_JUNCTION) | 132 | 0.52 | 2.17 | 0.000 | 0.001 | 0.008 | 7514 | tags=40%, list=14%, signal=46% |
| [KEGG_RENIN_ANGIOTENSIN_SYSTEM](http://www.gsea-msigdb.org/gsea/msigdb/cards/KEGG_RENIN_ANGIOTENSIN_SYSTEM) | 17 | 0.81 | 2.17 | 0.000 | 0.001 | 0.008 | 4446 | tags=65%, list=8%, signal=70% |
| [KEGG_GLIOMA](http://www.gsea-msigdb.org/gsea/msigdb/cards/KEGG_GLIOMA) | 65 | 0.57 | 2.16 | 0.000 | 0.001 | 0.010 | 7140 | tags=48%, list=13%, signal=55% |
| [KEGG_TGF_BETA_SIGNALING_PATHWAY](http://www.gsea-msigdb.org/gsea/msigdb/cards/KEGG_TGF_BETA_SIGNALING_PATHWAY) | 86 | 0.57 | 2.16 | 0.002 | 0.001 | 0.012 | 6624 | tags=50%, list=12%, signal=57% |
| [KEGG_SMALL_CELL_LUNG_CANCER](http://www.gsea-msigdb.org/gsea/msigdb/cards/KEGG_SMALL_CELL_LUNG_CANCER) | 84 | 0.57 | 2.09 | 0.000 | 0.002 | 0.026 | 7140 | tags=43%, list=13%, signal=49% |
| [KEGG_PROSTATE_CANCER](http://www.gsea-msigdb.org/gsea/msigdb/cards/KEGG_PROSTATE_CANCER) | 89 | 0.55 | 2.09 | 0.000 | 0.002 | 0.027 | 7140 | tags=46%, list=13%, signal=53% |
